# Probing the mechanism of cardiovascular drugs using a covalent levosimendan analog

**DOI:** 10.1016/j.yjmcc.2016.02.003

**Published:** 2016-03

**Authors:** Sandra E. Pineda-Sanabria, Ian M. Robertson, Yin-Biao Sun, Malcolm Irving, Brian D. Sykes

**Affiliations:** a Department of Biochemistry, Faculty of Medicine & Dentistry, University of Alberta, Edmonton, AB T6G 2H7, Canada; b Randall Division of Cell and Molecular Biophysics and British Heart Foundation Centre of Research Excellence, New Hunt's House, Guy's Campus, King's College London, London SE1 1UL, UK

**Keywords:** Troponin C, Levosimendan, Calcium sensitizer, Covalent modulator, Heart failure

## Abstract

One approach to improve contraction in the failing heart is the administration of calcium (Ca^2 +^) sensitizers. Although it is known that levosimendan and other sensitizers bind to troponin C (cTnC), their *in vivo* mechanism is not fully understood. Based on levosimendan, we designed a covalent Ca^2 +^ sensitizer (i9) that targets C84 of cTnC and exchanged this complex into cardiac muscle. The NMR structure of the covalent complex showed that i9 binds deep in the hydrophobic pocket of cTnC. Despite slightly reducing troponin I affinity, i9 enhanced the Ca^2 +^ sensitivity of cardiac muscle. We conclude that i9 enhances Ca^2 +^ sensitivity by stabilizing the open conformation of cTnC. These findings provide new insights into the *in vivo* mechanism of Ca^2 +^ sensitization and demonstrate that directly targeting cTnC has significant potential in cardiovascular therapy.

## Highlights

• A Ca^2 +^ sensitizer, i9 was designed that forms a covalent bond with C84 of cTnC.
• i9 stabilized the open state of the N-domain of cTnC.
• The structure of the covalent cTnC-cTnI-i9 complex was solved by NMR.
• The structure showed that i9 binds deep in the hydrophobic pocket of cTnC.
• Despite slightly reducing cTnI affinity, i9 enhanced the Ca^2 +^ sensitivity of cardiac muscle.

Decreased cardiac contractility is a characteristic of a variety of heart diseases including systolic heart failure. One approach to treating this condition is to administer positive inotropes that increase cardiac muscle contraction by increasing intracellular Ca^2 +^ concentration. Although beneficial in the short-term, over time this can lead to the development of arrhythmias and myocyte death due to Ca^2 +^ overload [1]. An emerging alternative is the administration of Ca^2 +^ sensitizers, a class of inotropes that act directly on the contractile proteins of the heart [1]. The best clinically characterized Ca^2 +^ sensitizer is levosimendan; however, its mechanism of action *in vivo* is still not fully understood. This work provides insight into the molecular mechanism behind Ca^2 +^ sensitization by characterizing the *in vitro* structure and *ex vivo* function of cTnC with a covalently bound levosimendan analog.

Contraction is regulated in the heart muscle by troponin (cTn) in a Ca^2 +^ dependent manner. cTn is a complex composed of C, I, and T subunits (cTnC, cTnI, and cTnT, respectively) localized to the thin filament of the sarcomere. cTnC contains two globular domains: the regulatory N-domain (cNTnC) that acts as the Ca^2 +^ sensor, and the structural C-domain that anchors cTnC on the thin filament. During systole, when the cytosolic Ca^2 +^ concentration increases, Ca^2 +^ binds to cNTnC and increases the prevalence of the open conformation of cNTnC [2], [3]. Following this conformational change, the switch region of cTnI (switch-cTnI) binds to cNTnC and drags the inhibitory and C-terminal regions of cTnI away from actin. This leads to an allosteric change in tropomyosin exposing the myosin binding sites on actin to promote the formation of the forc*e*-producing cross-bridges [4], [5].

One of the most widely studied Ca^2 +^ sensitizers is levosimendan. While levosimendan binds to cTnC *in vitro*, the details of its *in vivo* action are not clear. Besides Ca^2 +^ sensitization, levosimendan has also been shown to have vasodilatory, anti-inflammatory, and anti-apoptotic effects [6], [7]. Although levosimendan inhibits phosphodiesterase 3 (PDE3) at high concentrations, its positive inotropic effect is thought to be due to its interaction with cTnC and not to an increase in intracellular Ca^2 +^ [8], [9], [10], [11]. Previous NMR studies showed that in the presence of cTnI, levosimendan interacts only with the regulatory N-domain [12]. No three-dimensional structure has been determined due to the short lifetime of the cTnC·levosimendan complex. Using amide chemical shift mapping by NMR, Sorsa and coworkers found widespread chemical shift perturbations throughout the N-domain but could not determine a specific binding site [13]. Methionine methyl chemical shift perturbations, however, suggest levosimendan binds in the hydrophobic cleft of cNTnC [14].

Other studies have shown that C84 is essential for binding [14], [15]. In a study published by Kleerekoper and Putkey, levosimendan did not bind to the N-domain of cTnC when C84 was mutated to a serine [16]. The authors also reported no binding in the presence of C84; however it has been since shown that the sulfhydryl-containing reducing agent used in that study, dithiothreitol (DTT), reacts with levosimendan and prevents its binding to cNTnC [13], [14]. Levosimendan contains two nitrile groups that can undergo nucleophilic addition in the presence of thiol groups. We have hypothesized that levosimendan can form a reversible covalent bond with C84 of cNTnC (Ian M. Robertson et al. 2016, in preparation).

There have been a number of studies that indicate that small molecules that bind to cTnC also alter Ca^2 +^ sensitivity [17], [18], but it is not clear if this interaction is directly responsible for the increased contractility in the muscle. For example, EMD57033 was shown to bind to cTnC and increase Ca^2 +^ sensitivity and the force of contraction. However, after the structure of EMD57033 bound to cTnC was solved [19], it was shown that EMD57033 likely enhances contractility by stabilizing actomyosin cross-bridges rather than through its interaction with cTnC [20]. The lack of direct evidence for levosimendan binding to cTnC in cardiac muscle has also fueled the debate over whether levosimendan functions primarily as a Ca^2 +^-sensitizer [21], a PDE3 inhibitor [22] or by some combination of the two mechanisms [11]. The K^+^-channel activation and subsequent vasodilation effect of levosimendan [23] has further complicated characterization of its *in vivo* function [24].

**Fig. 1:**
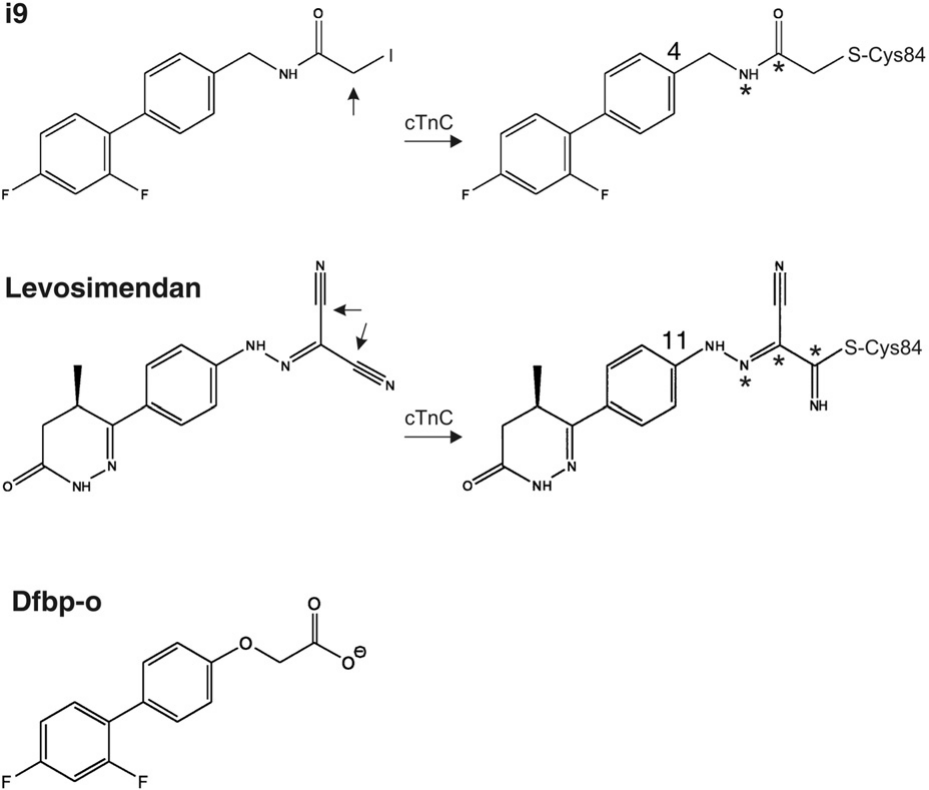
The covalent levosimendan-analog i9. Structures of i9, levosimendan, and dfbp-o. Arrows point to reactive carbon atoms. The structures of i9 and levosimendan reacted with C84 are shown. Carbons 4 and 11 are labeled to delimitate the spacer region between the biphenyl moiety and Sγ of C84. Atoms with trigonal planar geometry are marked with *.

To investigate whether a small molecule that we are certain is bound to cTnC can increase Ca^2 +^ sensitivity in cardiac muscle, we designed a novel levosimendan analog (i9) that covalently reacts with cTnC (Fig. 1) and replaced native cTnC with this complex in cardiac trabeculae. We studied the effect of i9 attached to cTnC(C35S) on contractility *ex vivo* and on switch-cTnI binding to cNTnC *in vitro.* We determined the NMR structure of a stable cNTnC·switch-cTnI hybrid protein (cChimera) with i9 covalently bound to C84. The results demonstrate that i9 increases the Ca^2 +^ sensitivity of contraction in cardiac muscle despite having a slightly reduced affinity of switch-cTnI for cTnC(C35S). This occurs because TnI is tethered to TnC in the muscle cell. Our structure shows that i9 binds in the hydrophobic cleft of cNTnC in close proximity to switch-cTnI. We conclude that i9 enhances muscle Ca^2 +^ sensitivity by stabilizing an open conformation of cNTnC. These findings provide new insights into the *in vivo* mechanism of Ca^2 +^ sensitization and demonstrate that directly targeting cTnC has significant potential as a cardiovascular therapy.

## 1 Results and discussion

### 1.1 Design of i9

To investigate the mechanism of action of levosimendan, we designed i9 based on the structures of levosimendan and its analog dfbp-o (Fig. 1). The three molecules contain a biphenyl group followed by a hetero-substituted moiety. The biphenyl group of dfbp-o was chosen because it was shown to insert in the hydrophobic cleft of cNTnC, conserve the Ca^2 +^ sensitization effect [25], and be advantageous for fluorine NMR [26]. The hetero-substituted moiety of i9 was designed based on the proposed reactivity of the nitrile group of levosimendan. A reactive iodoacetamide group was incorporated such that the number of bonds separating the biphenyl moiety and the sulfur atom of C84 was the same as with levosimendan (Fig. 1). The covalent analog i9 has a planar center on the amide N, which closely resembles levosimendan.

### 1.2 Synthesis and purification of i9

To synthesize the covalent levosimendan analog i9, we followed the route outlined in Supporting Figure 1. Supporting Figure 1b shows the ^1^H NMR spectra of **1**, **2**, and i9 in DMSO-*d*_6_. With the addition of the acetyl chloride moiety the signal corresponding to N8 is shifted downfield (from 8.36 to 8.75 ppm) as the result of deshielding by the newly attached carbonyl C9, also a new aliphatic signal at 4.35 ppm is observed corresponding to the new methylene protons at C10. The halogen exchange from Cl to I shifted the H10 singlet upfield (from 4.12 to 3.71 ppm) due to the less electronegative character of I compared to Cl. Both reactions had only small effects on the aromatic protons of the products, which were assigned with aid from previous assignment of dfbp-o [25].

### 1.3 Troponin labeling with i9

Three proteins were independently reacted with i9: cTnC(C35S) for physiological characterization, cNTnC for assessment of reaction specificity, and ^13^C,^15^N-cChimera for structure determination by NMR. cChimera is a hybrid protein which contains cNTnC (residues 1–89) and switch-cTnI (residues 144–173) that represents the cNTnC·switch-cTnI complex prevalent during the systolic state of the heart [27]. The labeling reactions were performed in urea or NMR buffer, and verified by ^19^F NMR and mass spectrometry.

**Fig. 2:**
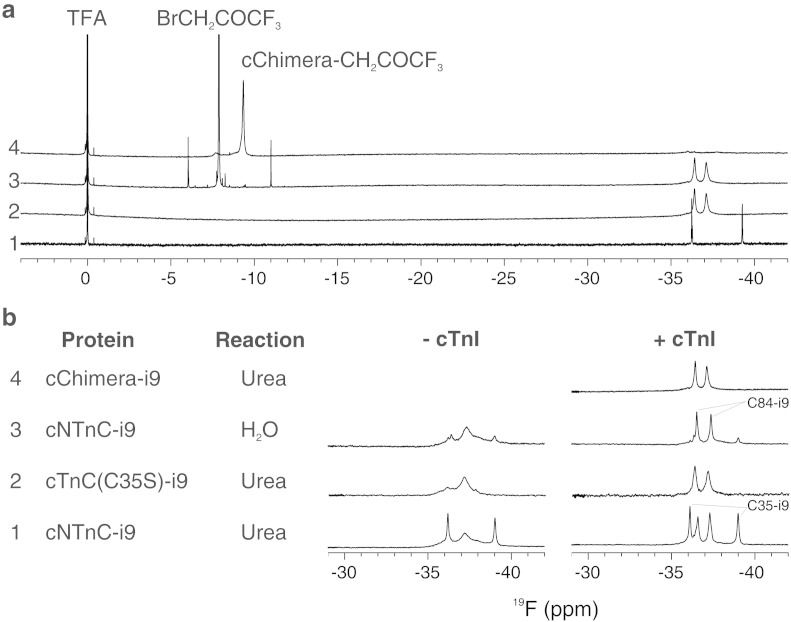
Troponin labeling with i9. a, stack of ^19^F spectra of i9 (1), cChimera-i9 (2), and cChimera-i9 in the presence of 3-bromo-1,1,1-trifluoroacetone (3) showing complete reaction of cChimera with i9. Spectrum 4 shows trifluoroacetone bound to cChimera for reference. b, stack of ^19^F spectra of cNTnC-i9, cTnC(C35S)-i9, and cChimera-i9 in the absence and presence of switch-cTnI. The reaction column specifies labeling conditions.

Protein labeling is illustrated for cChimera in Fig. 2a, where the ^19^F NMR spectrum of i9 (spectrum 1) shows two sharp signals at − 36.2 and − 39.3 ppm corresponding to the fluorine atoms F4′ and F2′. After the reaction with cChimera (spectrum 2) the two fluorine signals shift (to − 36.4 and − 37.1 ppm) and broaden as a result of a change in environment and molecular size, respectively. To assess the completion of the reaction, 0.2 mM-bromo-1,1,1-trifluoroacetone was added to cChimera-i9 to react with any remaining free sulfhydryl group present (spectrum 3). The presence of the unreacted trifluoroacetone only, as a sharp singlet at − 8 ppm, indicates that the reaction with i9 was complete. Spectrum 4 shows the chemical shift of trifluoroacetone bound to cChimera in a different sample for reference.

Fig. 2b displays a summary of the ^19^F spectra of all the reacted proteins. Full labeling of cNTnC with excess i9 in urea results in covalent binding at C35 and C84, but only the signals corresponding to C84-i9 sharpen in the presence of cTnI. The ^19^F spectrum of cNTnC-i9 in the absence of switch-cTnI (Fig. 2b, spectrum 1) shows two sharper signals corresponding to F2′ and F4′ of C35-i9, and broader signals for C84-i9, which indicates multiple conformations for C84-i9. Addition of a 3:1 excess switch-cTnI (residues 144–173), which binds to cNTnC, causes the fluorine signals of C84-i9 to sharpen. This indicates that binding of switch-cTnI to cNTnC stabilizes C84-i9 in one conformation. In a similar way, the ^19^F spectrum of cTnC(C35S)-i9 (spectrum 2) shows the same change in linewidth for F2′ and F4′ of C84-i9 upon addition of switch-cTnI. For cChimera-i9 (spectrum 4), in which switch-cTnI is bound to cNTnC, the C84-i9 signals are very similar to those of cTnC(C35S)-i9 and cNTnC-i9 in the presence of switch-cTnI. Comparable spectra indicate similar electronic environments for i9 in all cases. Thus, the i9 molecule is expected to adopt the same conformation in all cNTnC·switch-cTnI systems. This validates the use of cChimera for structural characterization of their interaction.

The reaction of i9 with cNTnC in aqueous NMR buffer using a small drug-to-protein excess (1.1:1) resulted in preferential labeling on C84 as judged by the appearance of intense C84-i9 peaks and minuscule C35-i9 peaks (spectrum 3). For an unspecific reaction, equal labeling of C35 and C84 would be expected. Selective labeling of C84 with i9 is most likely the result of a two-step process as is the case for covalent inhibitors [28]. Initially, non-covalent binding to the target protein positions the reactive groups close in space. Then the complex undergoes bond formation.

### 1.4 Ca^2 +^ sensitizing effect of cTnC(C35S)-i9

**Fig. 3:**
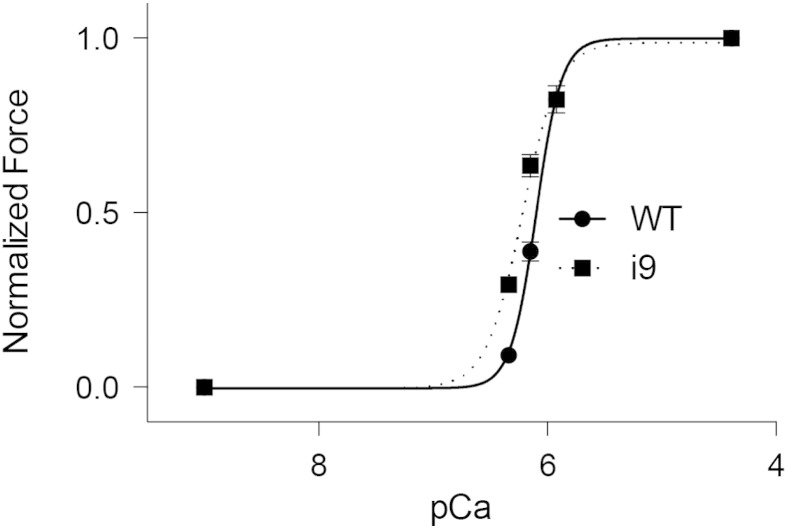
Effect of i9 on muscle fiber contraction. Forc*e*-pCa relationship of ventricular trabeculae containing wild type cTnC (WT) and cTnC(C35S)-i9 (i9). Data points are means ± SEM (*n* = 7).

We investigated the effect i9 had on contraction in demembranated ventricular trabeculae containing cTnC(C35S)-i9. Following the exchange of native cTnC for cTnC(C35S)-i9 in ventricular trabeculae, an increase in the Ca^2 +^-sensitivity of force development was observed (Fig. 3). The data were fitted with the Hill equation (Eq. (1)) and the pCa_50_ increased from 6.10 ± 0.01 (SEM; *n* = 7) to 6.22 ± 0.01 (SEM; *n* = 7) (*P* < 0.05). The maximum Ca^2 +^-activated isometric force was 25.3 ± 1.7 mN mm^− 2^ (SEM; *n* = 8) prior, and 24.6 ± 2.7 mM mm^− 2^ (SEM; *n* = 7) after exchange of cTnC. Thus, cTnC(C35S)-i9 did not affect maximum Ca^2 +^-activated force, which is consistent with the observations made for the Ca^2 +^-sensitizers dfbp-o [25] or levosimendan [29]. We also observed a decrease in the Hill coefficient (an indicator of cooperativity) from 4.62 ± 0.53 (SEM; *n* = 7) to 2.90 ± 0.34 (SEM; *n* = 7) following exchange. A similar decrease in cooperativity has been observed for other Ca^2 +^ sensitizers [30] as well as several Ca^2 +^-sensitizing mutations [31]. The amount of cTnC(C35S)-i9 exchanged within the muscle was estimated to be 20% by LC-MS. When a higher fraction of native cTnC in trabeculae was replaced by cTnC(C35S)-i9, active force developed in relaxing (pCa 9) conditions. This suggests that i9 stabilizes a conformation of cTnC similar to that stabilized by Ca^2 +^.

Because i9 is covalently attached to C84, the observed increase in Ca^2 +^ sensitivity can be unequivocally attributed to i9 binding to cNTnC. This is in contrast to traditional experiments in which muscle is soaked in solutions containing a drug under study [32], [33], [34], [35], [36] leaving the *in situ* target uncertain. Since cTnC shares many structural features with other contractile EF-hand regulatory proteins, such as the myosin regulatory and essential light chains [37], it is always a concern whether changes in contractility are due exclusively to binding to cTnC.

### 1.5 Conformation of cNTnC(C35S)-i9

**Fig. 4:**
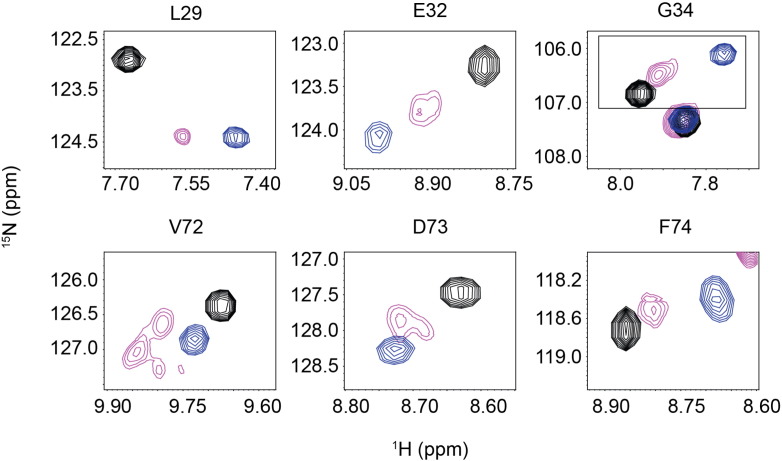
Conformation of cNTnC(C35S)-i9. Overlap of regions of the ^1^H,^15^N-HSQC NMR spectra of cNTnC (black) representative of the closed state, cNTnC·switch-cTnI (blue) representative of the open state, and cNTnC-i9 displaying an intermediate state (pink).

To investigate if i9 stabilizes the open conformation of cNTnC in the absence of switch-cTnI, we compared the ^15^N and ^1^H amide chemical shifts of cNTnC(C35S)-i9 with those of cNTnC in the absence and presence of switch-cTnI, which are characteristic of the closed and open states [38], respectively (Fig. 4). In most cases, resonances of cNTnC-i9 lie between those of cNTnC and cNTnC·switch-cTnI, suggesting cNTnC-i9 adopts a partly open conformation. Interestingly V72 is shifted further than the resonance indicative of the open conformation. Residues such as V72, D73, and E32 display more than one signal for their amide NH resonances indicative of multiple conformations. These results support our earlier supposition that i9 is in more than one conformation in the absence of switch-cTnI (Fig. 2b, spectra 1–3).

To quantify the predicted conformation of cNTnC-i9, we used ORBplus [38]. ORBplus uses amide chemical shifts to predict the AB and CD interhelical angles, which are good indicators of the overall conformation of cNTnC (angles closer to 90° indicate a more open conformation). Residues in or near Ca^2 +^ binding site I (27–40) are good indicators of the AB interhelical angle, and residues in or near site II (64–74) are good indicators of the CD interhelical angle [38]. Due to the presence of multiple peaks and exchange broadening for some cNTnC-i9 residues, it was not possible to obtain complete assignment of all residues in sites I and II. Using the amide chemical shifts of L29, G30, A31, E32, G34, and S35, the AB interhelical angle is predicted to be 133°, which is 9° more open than cNTnC (142°). Using resides E66, D67, G68, V72, D73 and F74, the CD interhelical angle is 105°, which is 4° more open than cNTnC (109°) (Table 1). These results indicate than i9 partially opens cNTnC and suggest that the AB-interhelical angle is more sensitive to i9 binding than is the CD interhelical angle. A similar magnitude of change (ΔAB = 10°; ΔCD = 5°) was observed for the Ca^2 +^-sensitizing mutation, L48Q [38], which suggests that altering the AB interhelical angle, even just slightly, can significantly increase Ca^2 +^-sensitivity.

### 1.6 Effect of i9 on switch-cTnI binding to cTnC(C35S)

**Fig. 5:**
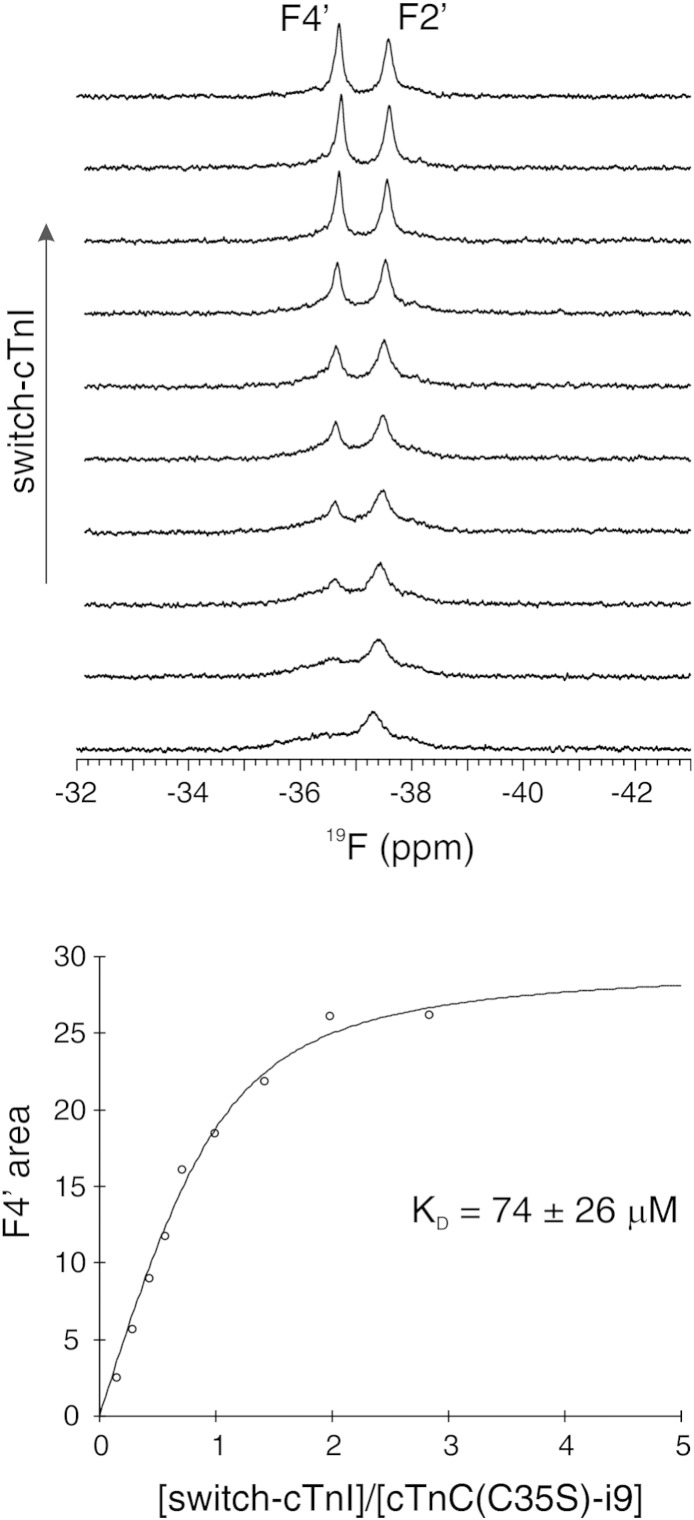
Effect of i9 on switch-cTnI binding to cTnC. Stack of ^19^F NMR spectra (top) and binding curve (bottom) for the titration of switch-cTnI into cTnC(C35S)-i9.

Along with the stabilization of the open state of cNTnC, enhanced switch-cTnI binding has been proposed to increase Ca^2 +^ sensitivity [25]. To evaluate the effect of i9 on switch-cTnI binding, we titrated Ca^2 +^-saturated cTnC(C35S)-i9 with switch-cTnI (residues 144–163) and monitored it by ^19^F NMR spectroscopy. The change of area under the F4′ signal of i9 as a function of increasing switch-cTnI concentration was fit to a binding curve with 1:1 stoichiometry and a dissociation constant (K_D_) of 74 ± 26 μM (SD, Fig. 5). This corresponds to an affinity approximately three times lower than that for the binding of switch-cTnI (residues 144–163) to cNTnC in the absence of i9 (K_D_ = 26 ± 4 μM (SD)) [25]. Likewise, the Ca^2 +^-sensitizer, bepridil, was also shown to reduce the affinity of switch-cTnI [39]. Thus, our results indicate the Ca^2 +^ sensitizing effect of i9, like bepridil, does not involve enhancing switch-cTnI binding. Interestingly, bepridil also impairs the cooperativity of contraction [30], and thus the reduced affinity of switch-cTnI may help explain the reduced cooperativity observed in the Ca^2 +^-sensitivity experiments [31].

Bepridil has been shown to enhance Ca^2 +^ affinity of cTnC [40], [41] and of various troponin complexes [42] through stabilizing the open conformation of cNTnC [43] and slowing the rate of Ca^2 +^ dissociation [40], [44]. Despite enhancing Ca^2 +^ affinity, bepridil actually increases the speed of transition from the open, cTnI-bound, conformation of cNTnC to the closed, cTnI dissociated, conformation of cNTnC [42]. This observation is probably due to the reduced affinity of switch-cTnI for the cNTnC-bepridil complex [39], [42], and seems to suggest that paradoxically, despite enhancing Ca^2 +^ affinity, bepridil may also promote diastolic relaxation [42]. Therefore, given the similar reduction in switch-cTnI affinity for cNTnC-i9, one may expect a similar enhancement of the relaxation rate of contraction as proposed for bepridil. It is important to note, as mentioned above, that substitution of > 20% of native cTnC with cTnC(C35S)-i9 led to active force generation even in the absence of Ca^2 +^. Therefore, although covalently bound i9 may promote the rate of cTnI dissociation, its prevention of complete relaxation limits its use as a treatment of heart failure.

On the other hand, levosimendan, which has also been shown to increase Ca^2 +^ affinity [15], [45], did not show the enhanced transition rate from an open conformation to a closed conformation that was observed for bepridil [42]. This suggests that levosimendan does not compete with switch-cTnI and that its mechanism for Ca^2 +^-sensitization may be different than bepridil's (and therefore also i9). However, in that study, C35 and C84 were mutated to serines in order to accommodate fluorophore labeling at other non-native cysteine residues [42]. Therefore the lack of C84, which is critical for levosimendan binding [14], [15], coupled with the relatively minor impact of levosimendan on Ca^2 +^-sensitivity [42], makes interpretation of this finding and how it applies to the mechanism of i9 unclear.

Finally, it is also possible that the 3-fold decrease in switch-cTnI affinity in the micromolar range (~ 25 to 75 μM) may not be significant in the context of the high apparent concentration of switch-cTnI in the thin filament. cNTnC and switch-cTnI are spatially confined in the thin filament such that the apparent concentration of switch-cTnI is high. We previously designed cChimera to mimic the *in situ* conditions of the thin filament. In this hybrid protein, cNTnC and switch-cTnI are tethered and the apparent concentration of switch-cTnI was determined to be ~ 1 mM [27]. Based on paramagnetic relaxation enhancement-NMR data, the Brown group also suggested that switch-cTnI remains in the vicinity of cTnC in the absence of Ca^2 +^ [46].

### 1.7 Structure of cChimera-i9

**Fig. 6:**
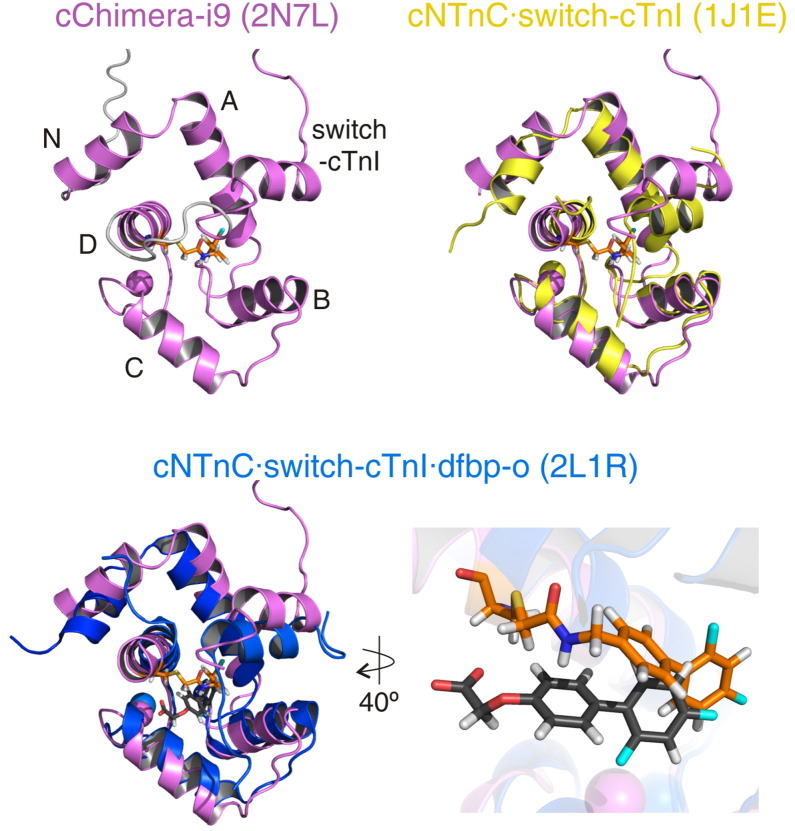
Structure of cChimera-i9. The lowest energy structure of cChimera-i9 (pink) shows the his-tag and linker region in grey. Helices on cNTnC are labeled N and A through D, the switch region of cTnI is labeled, i9 is in orange sticks, Ca^2 +^ ions are shown as spheres. The linker region is not shown in the overlays for clarity. The structures of cNTnC·switch-cTnI (yellow) and cNTnC·switch-cTnI·dfbp-o (blue) were aligned to cChimera-i9 using the alpha carbons of all helices. The expansion shows the positions of i9 and dfbp-o in the hydrophobic pocket of cNTnC

To characterize the interaction of a covalent Ca^2 +^ sensitizer with the regulatory cNTnC·switch-cTnI complex, we determined the structure of cChimera-i9. In cChimera, the high apparent concentration of switch-cTnI keeps the complex in a switch-cTnI-saturated state [27]. This design allows for structural assessment of cNTnC·switch-cTnI as it is found during systole in the heart. The structure of cChimera-i9 (Fig. 6; Supporting Figure 2) is similar to the structure of the cNTnC·switch-cTnI complex observed in other structures (see Table 1 for a list of interhelical angles). When cNTnC and switch-cTnI in cChimera-i9 are compared to those in the x-ray structure of the core domain of cTn [47], the rmsd of alpha carbons is 2.2 Å for all residues and 2.0 Å for helical residues. Compared to those in the NMR structure of dfbp-o bound to cNTnC·switch-cTnI, the rmsd of alpha carbons is 2.2 Å for all residues and 2.1 Å for helical residues. The structure has been deposited in the Protein Data Bank (PDB) and the Biological Magnetic Resonance Data Bank (BMRB) under the ID 2N7L and 25810, respectively. Structural statistics for the final ensemble are summarized in Supporting Table 1.

The typical structural features of cNTnC in the Ca^2 +^ and switch-TnI bound state are present in the structure of cChimera-i9. It contains five α-helices, N and A through D, and a small β-sheet involving the loops of each EF-hand. Helices N and D in cChimera-i9 are extended by 2 and 5 residues, respectively (D2-D3 and K86-E90). The linker region between cNTnC and switch-cTnI in cChimera remains flexible. We confirmed the flexibility of the linker using the random coil index (RCI) analysis performed within TALOS +, which estimates values of the model-free order parameter S^2^ based on the chemical shift of CA, CB, N, HA, and NH, backbone atoms [48]. The RCI indicates that residues 95 and 96 of the linker along with 144–147 of cTnI have S^2^ < 0.5 and are classified as dynamic.

### 1.8 Structure of cTnI in cChimera-i9

**Fig. 7:**
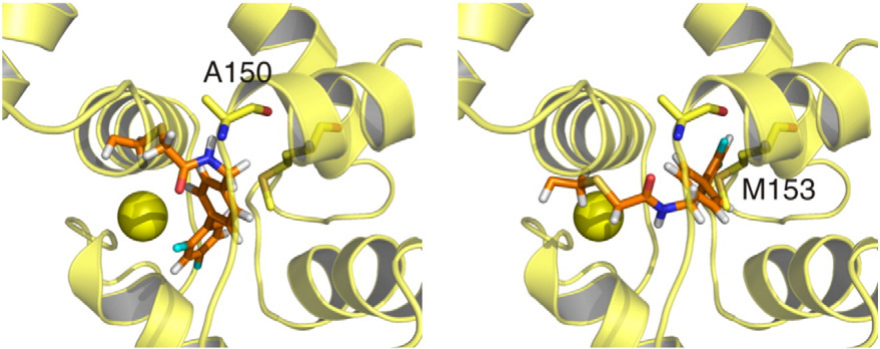
Potential steric clash between i9 and cTnI. The alpha carbons of cNTnC from the structure of cChimera-i9 and from the x-ray structure of the core domain of cTn (yellow) are aligned. Although both cNTnC and switch-cTnI from the x-ray structure are shown, only the i9 molecule and C84 from the structure of cChimera-i9 are shown (orange sticks). Two representative models from the final ensemble of cChimera-i9 are depicted: on the left is the lowest energy model and on the right is the model closest to the average model. Two residues from the x-ray structure that clash with i9 in each model are A150 and M153 (yellow sticks).

Switch-cTnI in cChimera-i9 forms an α-helix that is slightly shifted away from the core of the protein. The length and composition of the switch helix of TnI in cChimera-i9 is the same as that observed in the crystal structure of the troponin core domain (1J1E) [47], starting at residue A150 and continuing until residue L158 with the adjacent regions being unstructured. The switch helices of both structures are roughly parallel and localize between the A-B-D helices of cNTnC. One possible explanation for the shift in switch-cTnI position relative to cNTnC is a steric clash between i9 and A150, at the start of the switch-helix, and/or M153, which faces the hydrophobic cleft of cNTnC (Fig. 7). Despite this steric clash between switch-cTnI and i9, cNTnC is in an open conformation. The AB interhelical angle is 99° and the CD interhelical angle is 91°, which are similar to the interhelical angles measured for cNTnC bound to switch-cTnI (Table 1). Interestingly, this is in contrast to W7 and bepridil, both of which also compete with switch-cTnI binding. This difference may be due to the fact that both i9 and switch-cTnI are covalently bound to cNTnC in the cChimera structure.

### 1.9 Structure of i9 in cChimera-i9

**Fig. 8:**
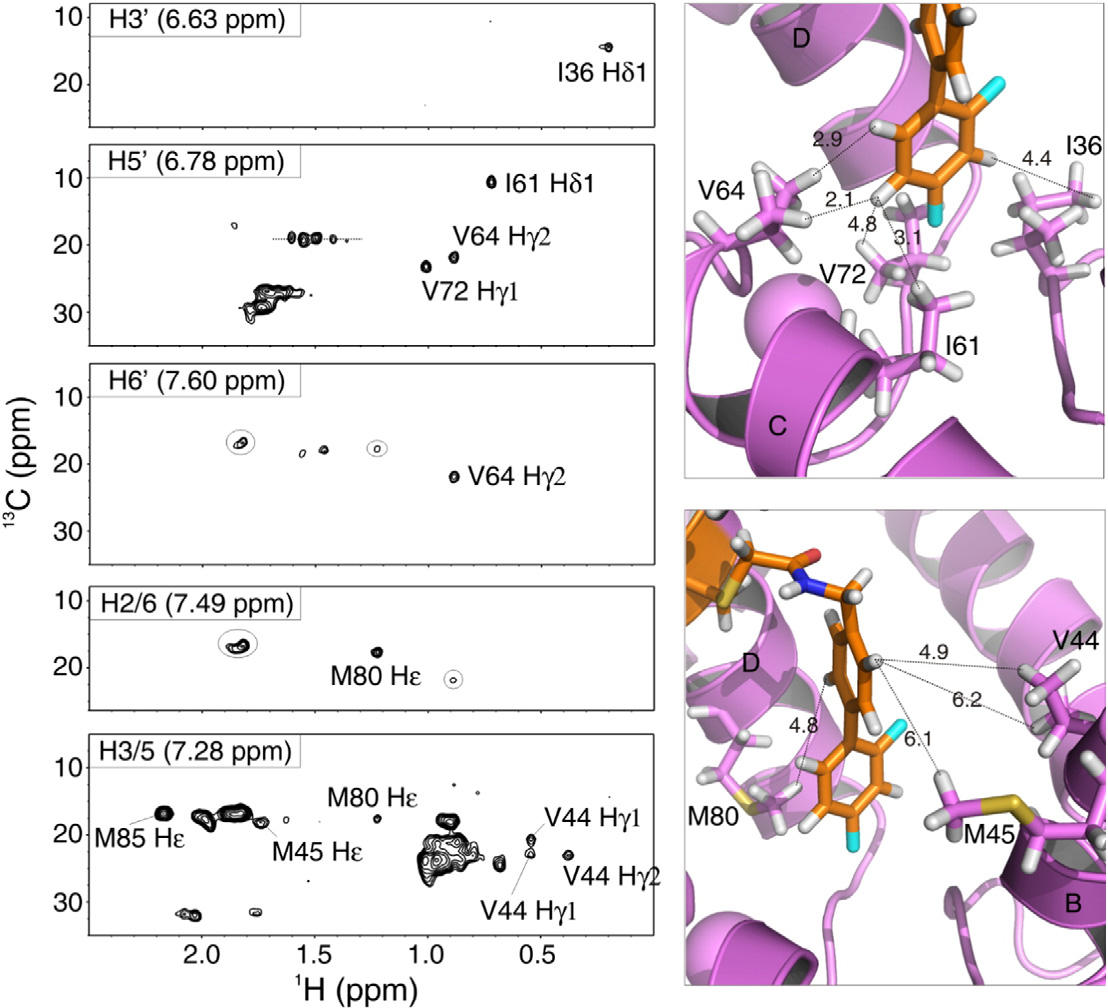
NOEs between i9 and cChimera. Planes from the three dimensional noesyChsqc_CNfilt NMR spectrum corresponding to each aromatic proton of i9 in cChimera-i9. The name and chemical shift of the i9 protons are labeled in the upper left corner of each plane. The NOEs to protons on cChimera (lowest energy structure) are also labeled and depicted in the panels on the right. In the panels, helices and residues of cNTnC (pink) are labeled, as well as the distances to i9 (orange) protons corresponding to the observed NOEs.

Fourteen NOE distance restraints define the position of i9 in the core of cChimera between helices B, C, and D of cNTnC, and the helical region of switch-cTnI. The difluorophenyl ring of i9 contacts residues I61 and V64 on helix C, and I36 and V72, which form part of the β-sheet of cNTnC (Fig. 8); this is consistent with its position deep in the hydrophobic cleft. The middle phenyl ring of i9 contacts several residues on the middle region of the cleft such as L41, V44, and M45 on helix C and M80 on helix D. This ring also makes NOEs to M85 on helix D and V146 on switch-cTnI, which are located towards the protein surface (Fig. 8).

The binding site of i9 is comparable to that of other drugs that interact with cTnC such as bepridil, dfbp-o, and W7 [25], [39], [49]. Compared to dfbp-o, i9 binds deeper in the pocket (Fig. 6). This indicates that the length of the hetero-substituted moiety of i9 is adequate to allow for deep binding (Fig. 1). Because this moiety of i9 was designed based on that of levosimendan, we propose that levosimendan binds in a similar fashion once it reacts with C84. The spacer between the biphenyl moiety of i9 and the reactive thiol of cNTnC (C4 of i9 to Sγ of C84, Fig. 1) has one double and four single bonds, the same as levosimendan would have once reacted with C84 (C11 of levosimendan to Sγ of C84, Fig. 1). However, the planarity of levosimendan in the spacer is extended compared to that of i9, which may slightly alter its conformation when bound.

Although no high-resolution structure of levosimendan bound to cTnC has been published, some studies have provided structural information about its binding site [14], [45]. In a complex between levosimendan and Ca^2 +^-saturated cNTnC (residues 1–91), NOEs between levosimendan and M85, M81 and F77 from cNTnC were tentatively assigned [45]. More recently, the ^13^C chemical shifts of methionine methyl groups from Ca^2 +^-saturated cTnC(C35S) were monitored by ^1^H, ^13^C-HSQC NMR spectroscopy before and after levosimendan binding [14]. The residues that experienced the largest chemical shift perturbations following levosimendan binding were M85, M81 and M47, suggesting that they are in close proximity to levosimendan [14]. In the cChimera-i9 structure in our study, i9 makes NOE contacts with M85, M80, and M45, which suggest that i9 and levosimendan have a similar binding site. It is worthwhile to note that the structural studies on levosimendan were done in the absence of cTnI; thus, the slight differences between studies may be the result of the presence of cTnI. For example, residues M81 and M47 lie at the interface formed between cNTnC and cTnI; therefore it is plausible that in the presence of cTnI, levosimendan would adopt a similar conformation as that seen for i9 in the cChimera complex.

**Fig. 9:**
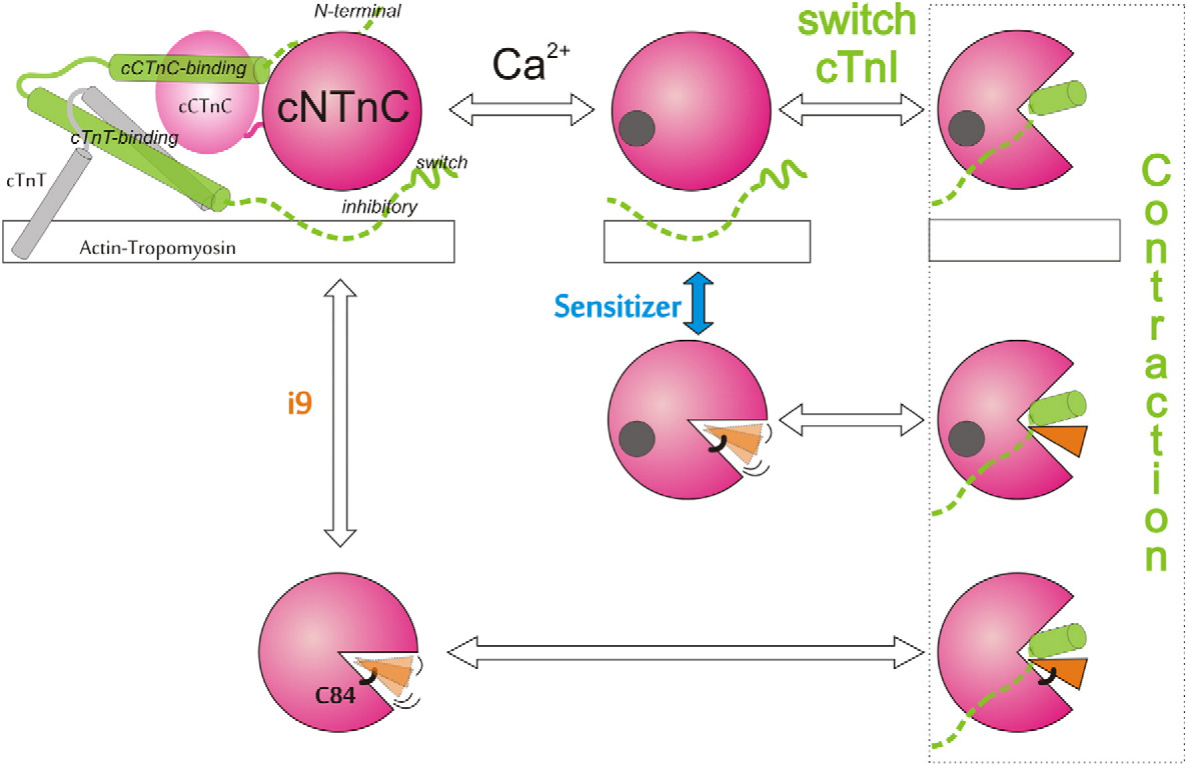
Proposed mechanism of action of i9 and other Ca^2 +^ sensitizers. i9 selectively binds to cNTnC, reacts with C84, and promotes the open state to induce positive inotropy. A Ca^2 +^ sensitizer may also bind to cNTnC and stabilize the open state without making a covalent bond. Only cNTnC and the inhibitory and switch regions of cTnI are shown after the first schematic for clarity.

We propose that i9 has a similar effect as Ca^2 +^ to enhance contraction (Fig. 9). cNTnC is in equilibrium between open and closed conformations. Ca^2 +^ binding to cNTnC shifts the equilibrium to the open state to allow the binding of switch-cTnI [2], [3], [50]. Our results indicate that the Ca^2 +^-sensitizer i9 is sufficient to turn on contraction, regardless of its effect on switch-cTnI binding. i9 may stabilize the open conformation either through shifting the equilibrium towards the open state or through preventing complete closure of cNTnC, even following Ca^2 +^ release. This can be extended to the mechanism of action of other Ca^2 +^ sensitizing agents that bind to cTnC. Details of the interaction of the sensitizers bepridil, dfbp-o, levosimendan, i9, and the desensitizer W7 with cNTnC are summarized in Table 2. All of these molecules favor the open state of cNTnC, regardless of their effect on Ca^2 +^ sensitization. This suggests that there is another downstream mechanism responsible for their differential effect on contractility. One possible explanation is that they alter the affinity of switch-cTnI for cNTnC (increased by Ca^2 +^-sensitizers and decreased by desensitizers). Although the effects of dfbp-o and W7 on switch-cTnI conform to this hypothesis, bepridil and i9 do not; both compete with switch-cTnI yet still enhance contraction. However, the decrease in switch-cTnI affinity is relatively minor when compared to W7 and is likely not physiologically relevant.

In conclusion, we have shown that if we ensure that the drug under study is bound to the designated target protein in the muscle, by covalently linking it to that protein and then exchanging the complex into the muscle, then it has the effect predicted on the basis of the *in vitro* mechanism. We did discover that the *in situ* mechanism overcomes one of the kinetic limitations of the *in vitro* mechanism from the co-localization of the proteins involved in the final conformational cascade that triggers contraction. We anticipate that this knowledge can lead the design of novel Ca^2 +^ sensitizers for cardiac muscle.

## 2 Methods

### 2.1 Purification of troponin

cTnC(C35S), ^13^C-^15^N-cChimera, and ^15^N-cChimera were expressed in *E. coli* as described elsewhere [51]. cChimera contains a histidine tag, a thrombin cleavage site (GGLVPRGS), cNTnC (residues 1–89), a TEV cleavage site (ENLYFQG), and switch-cTnI (residues 144–173). We previously showed that cChimera resembles the cNTnC·switch-cTnI complex in the ~ 74% bound state [27]. The cChimera proteins were purified by Ni-NTA affinity followed by gel filtration chromatography as previously reported [27]. ^15^N-cNTnC(C35S) was obtained by TEV cleavage of ^15^N-cChimera [27]. The DNA from cTnC(C35S, C84S) [52] was used as a template for the preparation of cTnC(C35S) using a sit*e*-directed mutagenesis kit and cTnC(C35S) was purified as previously described [53]. The purity of the proteins was verified by reverse-phase HPLC and electrospray ionization Mass Spectrometry (E*SI*-MS). The synthetic cTnI peptide (residues 144–173) was obtained from GL Biochem Ltd. (Shanghai, China).

### 2.2 Synthesis and purification of i9

Chloroacetyl chloride was from Fluka Analytical (≥ 99% GC), (2′,4′-difluorobiphenyl-4-yl) methanamine (97%, compound **1**) from Amatek Chemical, and ethyldiisopropylamine (Hunig's Base, HB) was from Sigma-Aldrich (99.5%). In a glass vial 50 μmol of **1** and 170 μmol of HB were dissolved in 1.2 mL of acetonitrile. In a separate glass vial, 500 μmol of chloroacetyl chloride were dissolved in 40 μL of acetonitrile and slowly added into the **1**/HB solution under the extraction hood. The reaction produced gas (HCl) and turned to pale yellow. Then 6 mL of water were added to produce compound **2** as a white floating solid. Compound **2** was washed with water, recovered by centrifugation, and dried under vacuum. The dry product **2** was then dissolved in 1 mL of acetone, and an excess (270 mg) of NaI previously dried for 2 h at 110 °C was added. The halogen exchange reaction proceeded overnight at 37 °C with color change to orange and production of bubbles and precipitation (NaCl). 5 mL each of ethyl acetate and H_2_O were added to the reaction in a separation funnel. The yellow organic phase was washed twice with water and once with 5% Na_2_S_2_O_3_ which turned the solution clear. The clear organic phase was collected and dried with anhydrous Na_2_SO_4_ until no clumps were observed. The final solution was evaporated, the i9 product redissolved in deuterated dimethyl formamide, aliquoted, and stored at − 20 °C wrapped in aluminum foil. The purity and identity of the products was verified by MS and NMR.

### 2.3 Troponin labeling with i9

Full cTnC(C35S), cNTnC (residues 1–89), and ^13^C,^15^N-cChimera were labeled under denaturing conditions. In addition, cNTnC was labeled in aqueous buffer to assess the specificity of the reaction. The denaturing buffer contained 6 M urea, 150 mM KCl, 50 mM TRIS, and 1 mM EGTA. The aqueous buffer consisted of 100 mM KCl and 10 mM imidazole at pH 8. The corresponding protein was dissolved in denaturing or aqueous buffer, 2 mM of fresh TCEP was added, and the solution incubated for 30 min to reduce cysteine residues. A stock solution of i9 in DMF-d_7_ was added in aliquots to the protein solution under stirring and the pH was readjusted to 8. The protein solution remained clear before the i9: protein ratio reached 1:1, after which the solution became turbid. The final ratio was > 2:1 for the reactions in urea and 1.2:1 for aqueous buffer. The reaction proceeded in the dark with constant stirring at 27 °C for 16 h. The reaction was stopped with four times excess DTT and spun down. The supernatant of the reaction in urea was applied to a size exclusion chromatography column to purify the labeled protein cTnC(C35S)-i9, cNTnC-i9, or cChimera-i9. The protein fraction was lyophilized and stored at 4 °C.

### 2.4 Animals

Male Wistar rats (200–250 g) were stunned and killed by cervical dislocation (Schedule 1 procedure in accordance with UK Animal (Scientific Procedures) Act, 1986). The hearts were quickly removed and rinsed free of blood in Krebs solution (Sigma-Aldrich, K4002) containing: 118 mM NaCl, 24.8 mM NaHCO_3_, 1.18 mM Na_2_HPO_4_, 1.18 mM MgSO_4_, 4.75 mM KCl, 2.54 mM CaCl_2_, 10 mM glucose, bubbled with 95% O_2_–5% CO_2_ for 30–60 min; pH 7.4 at 20 °C. Unbranched trabeculae (diameter < 250 μm) were dissected from the right ventricle in Krebs solution containing 25 mm 2,3-butanedione-monoxime. The trabeculae were permeabilized in relaxing solution (see below) containing 1% Triton X-100 for 30 min, stored in relaxing solution containing 50% (v/v) glycerol at − 20 °C for experiments, and used within 2 days of dissection.

### 2.5 Reconstitution of cTnC(C35S)-i9 into ventricular trabeculae

Demembranated ventricular trabeculae were mounted via aluminum T-clips between a force transducer (AE801) and a fixed hook in a 60 μl trough containing relaxing solution. The sarcomere length (SL) was set to 2.1 μm by diffraction pattern using a Helium-Neon laser (632.8 nm). Experimental solutions contained 25 mM imidazole, 5 mM MgATP, 1 mM free Mg^2 +^, 10 mM EGTA (except pre-activating solution), 0–10 mM total calcium, 1 mM dithiothreitol and 0.1% (v/v) protease inhibitor cocktail (P8340, Sigma). Ionic strength was adjusted to 200 mM with potassium propionate; pH was 7.1 at 20 °C. The concentration of free Ca^2 +^ was calculated using the program WinMAXC V2.5 (http://web.stanford.edu/~cpatton/maxc.html). The calculated free Ca^2 +^ concentration was in the range 1 nM (pCa 9) to 41 μM (pCa 4.39). In pre-activating solution, the concentration of EGTA was 0.2 mM and no calcium was added. For all experiments, the temperature was 20–22 °C.

Trabecular activation was preceded by a 1 min incubation in pre-activating solution. Isometric force was measured after steady-state force had been established at each Ca^2 +^ concentration. Maximum force was recorded before and after each series of activations at submaximal Ca^2 +^ concentration. If the maximum force decreased by > 15%, the trabecula was discarded. The dependence of force on [Ca^2 +^] was fitted to data from individual trabeculae using non-linear least-squares regression to the Hill equation

(1) $$Y=\frac{1}{\left({1+10^{n_{H}}}^{\left(\mathrm{pCa}-{\mathrm{pCa}}_{50}\right)}\right)}$$

where *pCa*_*50*_ is the *pCa* corresponding to half-maximal change and *n*_*H*_ is the Hill coefficient. All values are given as mean ± standard error of the mean except where noted, with n representing the number of trabeculae.

Following initial characterization of Ca^2 +^-dependent cardiac muscle contraction containing native cTnC, cTnC was partially replaced by incubating the mounted trabeculae in relaxing solution containing 30 μmol/L cTnC(C35S)-i9 for 15 min at 20–22 °C. The muscle was subsequently washed 2–3 times in relaxing solution (without cTnC(C35S)-i9) and the Ca^2 +^-dependent cardiac muscle contraction was measured. If the SL had changed during the exchange, it was re-set to 2.1 μm.

The fraction of TnC replaced by cTnC(C35S) was estimated to be approximately 20% using LC-MS. Briefly, following the cTnC(C35S)-i9 exchange, the trabeculae were incubated for 1 h in 50 mM BDM, 25 mM Tris (pH 8.4) and 5 mM CDTA to extract all cTnC (native and cTnC(C35S)-i9). Due to the low concentration of cTnC, the extraction solution from three muscle fibers were combined and concentrated in a 3 K Amicon Ultra Tube. The solution was loaded on a Hewlett Packard (Agilent) 1100 Series LC/MSD using the electrospray ionization method and detected in positive mode. The spectrum was deconvoluted using the Agilent ChemStation software with an abundance cutoff set to 40%.

### 2.6 NMR spectroscopy

The NMR samples consisted of 0.3–0.8 mM cTnC(C35S)-i9, cNTnC-i9, cChimera-i9, or cNTnC(C35S)-i9 in 500 or 600 μL of 100 mM KCl, 10 mM imidazole or imidazole-d_4_, 2 mM CaCl_2_, and 0.25 mM 2,2-dimethyl-2-silapentane-5-sulfonate-*d*_6_ sodium salt (DSS-*d*_6_) or trifluoroacetic acid (TFA) as internal reference, at pH 6.9 (NMR buffer). The NMR experiments were acquired in 500, 600, or 800 MHz Varian spectrometers at 30 °C. All one-dimensional experiments were processed with VnmrJ v 3.2, all the multidimensional spectra were processed with NMRPipe [54] and analyzed with NMRViewJ [55]. The assignment of free i9 in DMSO was done based on examination of the ^1^H NMR spectra acquired throughout the synthesis, and on previous assignment of the levosimendan analog dfbp-o [25]. The assignment of i9 in cChimera-i9 was achieved using the ^13^C, ^15^N filtered noesy, ^13^C, ^15^N filtered tocsy, and ^1^H, ^19^F HMQC spectra (Supporting Figure 3 and Supporting Table 2). Assignment of cChimera in cChimera-i9 was done by using typical 2d and 3d NMR experiments ^1^H, ^15^N- and ^1^H, ^13^C-HSQC, HNCACB, CBCA(CO)NH, HNHA, HCCONH, and CCONH detailed in Supporting Table 2.

### 2.7 Switch-cTnI titration into cTnC(C35S)-i9

A solution of 530 μM cTnC(C35S)-i9 in NMR buffer was titrated with increasing amounts of switch-cTnI (residues 144–163) using a stock solution of 10.6 mM in DMSO-*d*_6_. The concentration of the protein solution was determined by amino acid analysis. The concentration of the stock solution was determined by NMR spectral integration of the methyl signals relative to that of a DSS-*d*_6_ standard. The concentration of switch-cTnI at each titration point was 0, 86, 169, 251,334, 415, 575, 810, 1,115, and 1,540 μM. The diluting effect of each switch-cTnI addition was taken in consideration when calculating the concentration of protein and peptide at each titration point. ^1^H and ^19^F NMR spectra were acquired after each addition of switch-cTnI. The change of area under the F4′ signal of i9 as a function of the switch-cTnI/cTnC(C35S) ratio was fit using a one-to-one stoichiometry with xcrvfit (www.bionmr.ualberta.ca/wiki/index.php/Main_Page).

### 2.8 Structure determination

The structure of i9 bound to cChimera was determined using Xplor-NIH v. 2.35 with experimental backbone dihedral and distance restraints. Parameter and topology files for i9 covalently attached to cysteine were generated using the PRODRG server [56] (http://davapc1.bioch.dundee.ac.uk/cgi-bin/prodrg/). The dihedral angles φ and ψ were predicted with the Talos + server (http://spin.niddk.nih.gov/bax/nmrserver/talos/) based on the chemical shift of HN, N, CA, CB, and HA backbone atoms of cChimera-i9. Intramolecular distance restraints within the protein component of cChimera-i9 were obtained from noesyNhsqc and noesyChsqc NMR spectra, NOEs were calibrated using the bin method of NMRViewJ and classified as strong (1.8–3 Å), medium (3–4.5 Å), and weak (4.5–6 Å). Intramolecular NOEs within i9 were obtained from the ^13^C, ^15^N filtered noesy spectrum in which signals from the ^13^C, ^15^N labeled protein moiety are filtered out to obtain NOEs from the unlabeled drug moiety only. Pseudo-intermolecular NOEs between cChimera and i9 were obtained from the three-dimensional noesyChsqc_CNfilt NMR spectrum; all of these were classified as weak (1.8–6 Å). We used statistical torsion angle potential to improve the quality of backbone and side chain conformations; this is based on over a million residues from high quality crystal structures from the PDB. We also used the gyration volume potential term to restrain the volume associated with the gyration tensor also based on values observed in the PDB. We used the anneal protocol of Xplor-NIH to generate 140 structures from which the lowest energy structure was used in the subsequent refine protocol. The final ensemble consists of the 20 lowest energy structures generated in the refinement step with no NOE violations > 0.4 Å or dihedral violation > 5°. This ensemble was validated with PROCHECK using the Protein Structure Validation Suite (PSVS 1.5) server (http://psvs-1_5-dev.nesg.org/).

## Figures and Tables

**Table 1:**
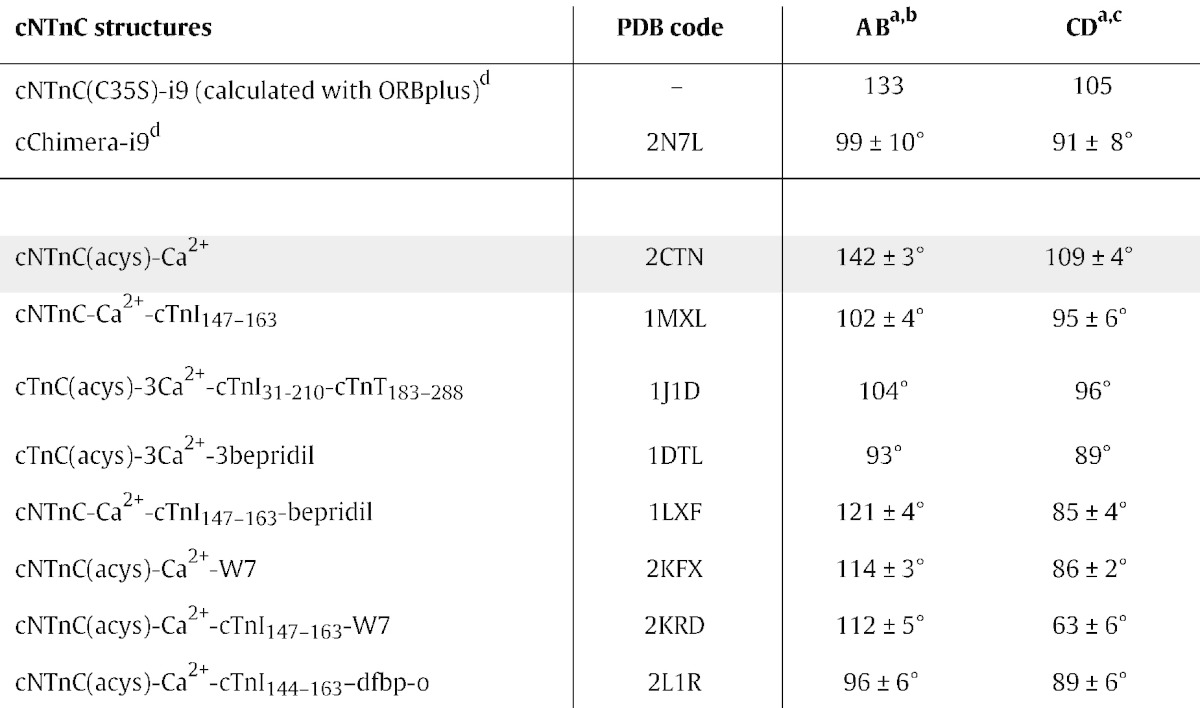
Inter-helical angles of cNTnC structures.^a^ The smaller the interhelical angle, the more open the cNTnC structure is. The reference closed state of cNTnC used by ORBplus is highlighted in grey.

^a^Angles calculated with the program Interhlx (K. Yap, University of Toronto).

^b^AB interhelical angles were determined by defining the C helix as residues 17–26, and the D helix was defined as residues 40–46 for all structures.

^c^CD interhelical angles were determined by defining the C helix as residues 54–62, and the D helix was defined as residues 75–83 for all structures.

^d^This work.

**Table 2:** Summary of drug binding to cTnC.

Drug molecule	Effect on Ca^2 +^ sensitization	Conformation of cNTnC	K_D_ (μM) cTnC/cTnC·cTnI	Localization of switch-cTnI	Affinity of switch-cTnI
Bepridil	Sensitizer	Open	23/80	Shifted away from cNTnC	~ 3.5-fold decrease
Dfbp-o	Sensitizer	Open	820/380	Same as cNTnC·cTnI	~ 2.2-fold increase
Levosimendan	Sensitizer	–	−/~200	–	–
i9	Sensitizer	Open	Covalent	Shifted away from cNTnC	~ 3-fold decrease
W7	Desensitizer	Open	~ 230/500	Shifted away from cNTnC	~ 13-fold decrease

## Appendix A Supplementary data

Supplementary figures and tables

Coordinates for cChimera-i9
