# Supplementary material for: Probing the mechanism of cardiovascular drugs using a covalent levosimendan analog
Source: J Mol Cell Cardiol. 2016 Mar;92:174–84. doi: 10.1016/j.yjmcc.2016.02.003 (PMC4831045; doi:10.1016/j.yjmcc.2016.02.003)
Supplement: Supplementary file 1 — Supplementary figures and tables [file mmc1.docx]

Supporting figures and tables


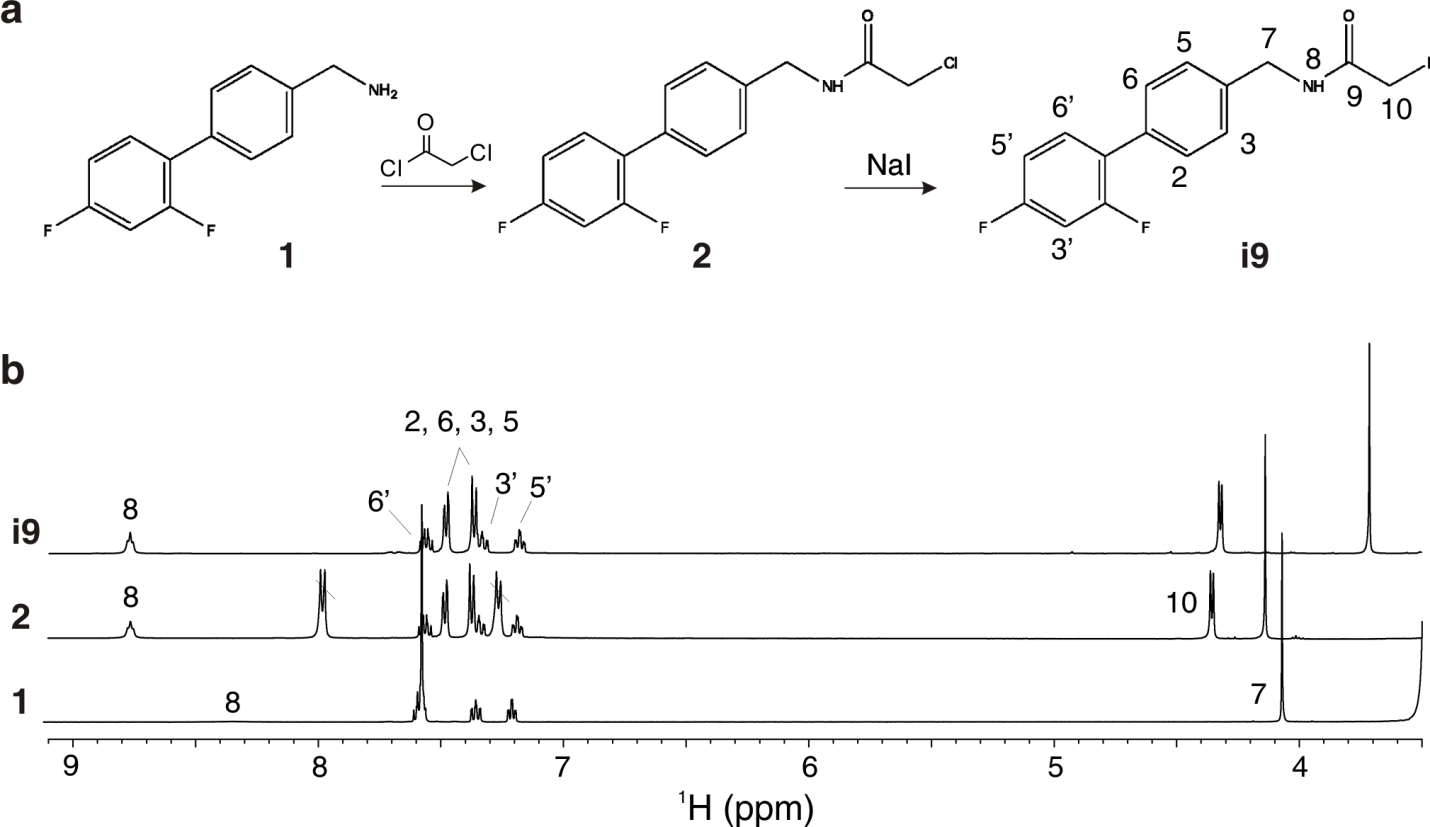


Supporting Figure 1. Synthesis of i9. a, synthetic route for the production of i9. b, stack of ^1^H NMR spectra in DMSO-d_6_ corresponding to each compound on the synthesis shown in a.


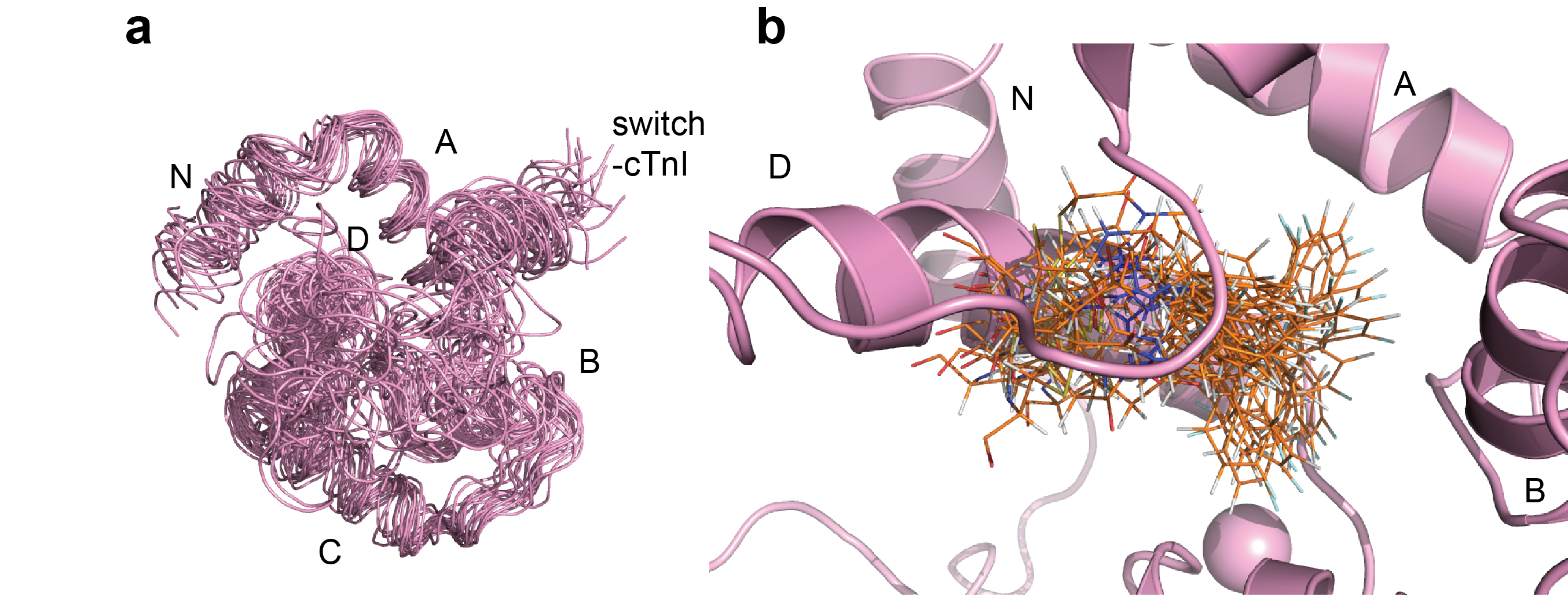


**Supporting Figure 2. Ensemble of the 20 lowest energy structures of cChimera-i9. a.** The ensemble of cChimera-i9 with the backbone from the his-tag, the c-terminus of cTnI, and i9 hidden for clarity. **b.** The ensemble of i9 (orange lines) is shown with only the lowest energy structure of cChimera depicted. The helices of cNTnC are labeled N and A though D and the switch region of cTnI is also labeled in both panels.


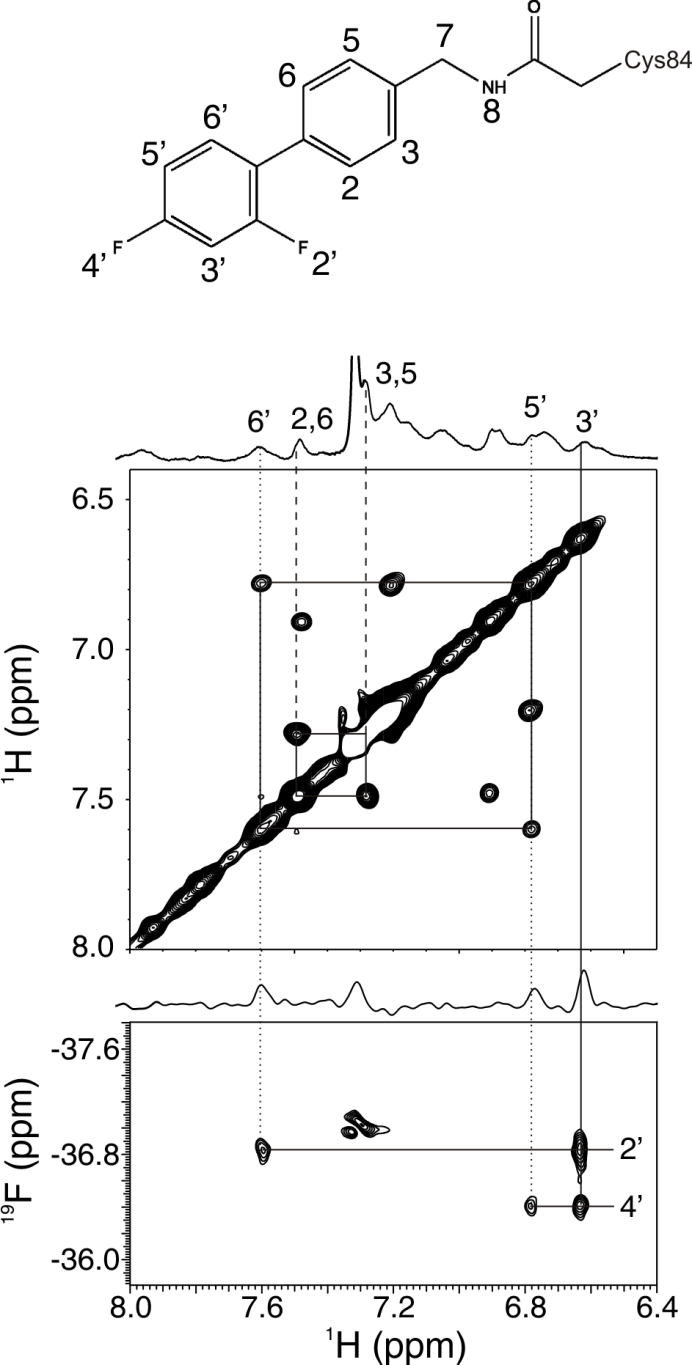


Supporting Figure 3. Assignment of i9 in cChimera-i9.

^13^C, ^15^N filtered noesy and ^19^F, ^1^H HMQC used to assign the resonances of aromatic protons of i9 when bound to cChimera. The chemical structure of i9 is shown for reference.

**Supporting Table 1. NMR and refinement statistics for the structure of cChimera-i9**

|  | **cChimera-i9** |
| --- | --- |
| Distance constraints |  |
| Total NOE | 1284 |
| Intra-residue | 794 |
| Inter-residue | 490 |
| Sequential (\|*i* – *j*\| = 1) | 275 |
| Medium-range (\|*i* – *j*\| < 4) | 116 |
| Long-range (\|*i* – *j*\| > 5) | 99 |
| Intermolecular | 0 |
| Hydrogen bonds | 0 |
| Total dihedral angle restraints | 99 |
| φ | 49 |
| ψ | 50 |
|  |  |
| Ramachandran statistics* |  |
| Most favored regions | 99.4 % |
| Additionally allowed regions | 0.6 % |
| Disallowed regions | 0 % |
|  |  |
| Average pairwise r.m.s. deviation* (Å) |  |
| Heavy | 2.3 |
| Backbone | 1.9 |

*For ordered residues 16-25, 28-42, 52-62, 68-78, 88-104, and 117-125 among 20 refined structures.

Supporting Table 2. Acquired NMR spectra for the study of cChimera-i9.

| Experiment name (Varian) | Solvent | Nucleus in  x/y/z  dimension | ^1^H  frequency  (MHz) | nt^a^ | x-points^b^ | y-points^b^ | z-points^b^ | x-sw^c^ | y-sw^c^ | z-sw^c^ | Mixing  time  (ms) |
| --- | --- | --- | --- | --- | --- | --- | --- | --- | --- | --- | --- |
| For assignment of i9 bound to cChimera | | |  |  |  |  |  |  |  |  |  |
| CNfilnoesy | D_2_O | ^1^H/^1^H | 600 | 64 | 4096 | 512 |  | 8398 | 8398 |  | 75 |
| CNfiltocsy | D_2_O | ^1^H/^1^H | 600 | 64 | 4096 | 256 |  | 8398 | 8398 |  | 43 |
| ^1^H ^19^F-HMQC | D_2_O | ^1^H/^19^F | 600 | 256 | 2048 | 56 |  | 8399 | 2500 |  |  |
| For assignment of cChimera bound to i9 | | |  |  |  |  |  |  |  |  |  |
| gNhsqc | H_2_O | ^1^H/^15^N | 600 | 4 | 512 | 128 |  | 8398 | 2432 |  |  |
| gChsqc | H_2_O | ^1^H/^13^C | 600 | 8 | 512 | 256 |  | 8398 | 10558 |  |  |
| ghn_cacb | H_2_O | ^1^H/^13^C/^15^N | 600 | 16 | 512 | 64 | 32 | 8398 | 12069 | 2432 |  |
| gcbca_co_nh | H_2_O | ^1^H/^13^C/^15^N | 600 | 16 | 512 | 64 | 32 | 8398 | 12069 | 2432 |  |
| ghnha | H_2_O | ^1^H/^1^H/^15^N | 600 | 8 | 512 | 128 | 32 | 8398 | 7198 | 1824 |  |
| ghc_co_nh | H_2_O | ^1^H/^1^H/^15^N | 600 | 16 | 512 | 72 | 40 | 8398 | 8398 | 2432 |  |
| gc_co_nh | H_2_O | ^1^H/^13^C/^15^N | 600 | 16 | 512 | 64 | 36 | 8398 | 12067 | 2432 |  |
| For structure determination | |  |  |  |  |  |  |  |  |  |  |
| gnoesyNhsqc | H_2_O | ^1^H/^1^H/^15^N | 600 | 8 | 512 | 128 | 48 | 8398 | 7198 | 1823 | 75 |
| gnoesyChsqc | D_2_O | ^1^H/^1^H/^13^C | 600 | 16 | 512 | 64 | 64 | 8398 | 5999 | 4525 | 100 |
| gnoesyChsqc_CNfilt | H_2_O | ^1^H/^1^H/^13^C | 800 | 16 | 820 | 64 | 48 | 9592 | 9596 | 16089 | 150 |

^a^ Number of scans,  ^b^ number of complex points,  ^c^ spectral width (Hz).
